# Factors affecting community ambulation post-stroke: a mapping review protocol

**DOI:** 10.12688/f1000research.144582.2

**Published:** 2024-08-29

**Authors:** Kanika Bansal, Jane Morgan-Daniel, Sudeshna A. Chatterjee, Dorian K. Rose

**Affiliations:** 1 Physical Therapy, University of Mount Union, Alliance, Ohio, USA; 2 Physical Therapy, University of Florida, Gainesville, Florida, USA; 3 Health Science Center Libraries, University of Florida, Gainesville, Florida, USA; 4 Department of Physical Therapy and Rehabilitation Sciences, Drexel University, Philadelphia, Pennsylvania, USA; 5 Brain Rehabilitation Research Center, Malcolm Randall VA Medical Centre, Gainesville, FL, USA; 6 Department of Physiology and Aging, University of Florida, Gainesville, Florida, USA; 7 Brooks Rehabilitation Clinical Research Centre, Jacksonville, FL, USA

**Keywords:** Real-world, Participation, Re-integration, Outdoor, Walking, Scoping Review

## Abstract

**Background:**

Most stroke survivors consider community ambulation an essential but unmet goal of their recovery. Historically, interventions to enhance community ambulation have focused on improving biomechanical impairments of gait; however, recent evidence suggests that biopsychosocial and environmental factors may impact community ambulation, even beyond more obvious physical impairments. The identification of factors that pose as significant facilitators or barriers to community ambulation may serve to guide stakeholders in designing relevant and evidence-based interventions for improving community ambulation post-stroke.

**Objective:**

This review aims to map the type and extent of existing evidence on the physical, biopsychosocial, and environmental factors affecting community ambulation post-stroke. Additionally, this review will describe the various methods used to examine the extent to which stroke survivors are restricted to community ambulation.

**Methods:**

Nine databases will be searched including CINAHL, PubMed, and Web of Science. We will include studies published in English during or after 2001. Studies that examine physical, biopsychosocial, and/or environmental factors affecting community ambulation in ambulatory adults at least six months post-stroke will be considered for inclusion. Studies that assess general physical activity or community mobility through transportation modes other than walking will be excluded. All identified records will be collated in citation management software, followed by steps of deduplication, title/abstract screening, and full-text reviews by at least two independent reviewers. The bibliographies of the extracted studies will also be reviewed for relevant articles. The extracted studies will be analyzed, critically appraised, and presented in tabular, narrative, and evidence map formats.

**Discussion:**

The evidence gained will be used to build a framework for community ambulation, informing stakeholders to develop meaningful interventions to improve community ambulation. The mapped evidence will motivate future studies to develop holistic approaches that specifically focus on the most vital factors that influence post-stroke community ambulation.

## Introduction

Stroke is a leading cause of chronic disability in the world. ^1^ In 2019, there were more than 101.5 million stroke survivors across the globe. ^1^ Poststroke muscle weakness and loss of voluntary motor control are associated with reduced gait speed, decreased endurance, and higher energy costs of walking. Indeed, adults post-stroke tend to spend more than 80% of their time sedentary, taking an average of 4,078 steps per day, far less than the recommended 6,500-8,500 steps per day for people with chronic disabilities. ^2^ With advances in gait rehabilitation, 65–85% of stroke survivors regain the ability to walk short distances independently, although they are limited to safe, stable, and structured environments such as their own homes or clinics. ^3^ More than 75% of stroke survivors consider their ability to ‘get out of the home’ as essential to very important for their recovery, ^4^ yet only 30% of them are satisfied with their level of community ambulation. ^5^ Community ambulation is most widely defined as “independent mobility outside the home, which includes the ability to confidently negotiate uneven terrain, private venues, shopping centers, and other spaces”. ^4^ The ability to walk safely in the community provides stroke survivors hope for recovery, a sense of independence, and a return to pre-stroke lifestyle, and may provide increased opportunities for social connections and overall engagement in life roles. ^6^ Participation in community ambulation may also help interrupt the commonly observed sedentary lifestyle post-stroke and may assist in reducing the risk of a second stroke. ^1^ Collectively, evidence suggests that improving community ambulation is a major but unmet goal of post-stroke gait rehabilitation.

Historically, most post-stroke physical therapy interventions focus on improving the physical, biomechanical, and neurological aspects of walking that lead to enhanced gait outcomes in the clinic, but have limited transferability to walking in a complex community environment. ^7^^,^^8^ However, since the emergence of the World Health Organization’s International Classification of Functioning, Disability, and Health (ICF) model in 2001, there has been a shift in focus from targeting only physical impairments to focusing on personal, environmental, and social factors impacting a person with stroke. Numerous studies have indicated that factors such as balance self-efficacy, ^9^ motivation, social support, ^10^ socioeconomic status, cognitive abilities, ^11^^,^^12^ and environmental terrains ^13^ impact community ambulation post-stroke, even beyond physical gait impairments. Recent studies indicate that interventions to improve community ambulation have not been successful unless they are accompanied by behavioral ^14^ or social change components. ^6^ However, thorough, and comprehensive guidance on which physical, personal, environmental, and social factors are significant facilitators or barriers to community ambulation is lacking. The multitude of factors impacting community ambulation also makes comprehensive assessment of community ambulation an ongoing challenge for clinicians. ^15^ Currently, the measurement of community ambulation includes a wide range of assessments, such as clinical outcomes, ^16^ self-report measures (surveys, diaries, and questionnaires), ^17^ and records of daily stepping activity through accelerometry. ^18^ A lack of consistent and uniform measurements of community ambulation is a major barrier to compiling results from existing clinical trials to guide future interventions. ^8^ Identifying tools that accurately examine the extent to which stroke survivors are restricted in their community ambulation is necessary for the development of evidence-based rehabilitation.

Based on the essential but unmet need to develop a comprehensive understanding of factors reported to impact post-stroke community ambulation, a mapping review methodology was deemed necessary. ^19^ The overarching objective of this review is to map the type and extent of the physical, biopsychosocial, and environmental factors affecting community ambulation in adults with chronic stroke and to describe the various tools used to determine the extent to which stroke survivors are restricted in their community ambulation. Specifically, this review will focus on individuals at least six months post-stroke to mitigate any confounding effects of post-stroke sequalae in the acute or subacute stage as stroke survivors transition from acute in-patient rehabilitation services to their homes, get used to their ‘new normal,’ and develop resilience to live their life after stroke. ^10^^,^^20^ Mapping reviews are subsets of scoping reviews; therefore, this project is guided by the Preferred Reporting Items for Systematic Reviews and Meta-Analyses extension for Scoping Reviews (PRISMA-ScR). ^19^^,^^21^ The methodology is also informed by the Campbell guidance for producing evidence and gap maps, which recommends conducting critical appraisal of included studies so that both the quantity and quality of the evidence can be recorded. ^22^

Previously, Wesselholf and colleagues conducted a systematic review focused on community mobility post-stroke, that is, accessing the community by any means of transportation, including walking, driving, bicycling, and using public transportation. ^23^ This systematic review concluded that community mobility is severely restricted in stroke survivors and may be affected by numerous factors such as age, education, general well-being, emotional state, motor function and coordination, independence in activities of daily living, balance, endurance, and driving status. ^23^ While community mobility can indeed occur with a wheelchair, ^24^ community ambulation requires walking outside one’s home or clinical environment ^17^ and, as discussed earlier, has numerous advantages for stroke survivors’ physical, mental, and social health and overall recovery. ^6^^,^^25^ This mapping review seeks to specifically identify reported factors impacting community ambulation post-stroke, which may differ from factors influencing community access by means of transportation other than walking. We conducted a preliminary search of BioMed Central Systematic Reviews, Campbell Collaboration Library (now known as Campbell Systematic Reviews), Cochrane Database of Systematic Reviews, JBI Database of Systematic Reviews (now JBI Evidence Synthesis), and PROSPERO: International Prospective Register of Systematic Reviews (November 15, 2018) and found no current or in-progress scoping, mapping, or systematic reviews on the proposed topic of factors affecting community ambulation post-stroke.

## Review question(s)

This mapping review aims to answer the following questions:

(i) Which physical, biopsychosocial, and environmental factors affect community ambulation in individuals post-stroke are reported in the literature and to what extent?
(ii) What are the various tools and clinical classification models used to measure or predict post-stroke community ambulation?
(iii) What does evidence suggest is the extent to which stroke survivors return to community ambulation?

## Inclusion criteria

### Participants

This review will consider studies that include community-dwelling individuals at least six months after their first-ever or recurrent stroke, who are at least 18 years of age and do not use a wheelchair as their primary mode of transportation. The use of assistive devices such as canes, walkers, or ankle-foot orthoses is permissible. We will exclude studies that include non-stroke participants or participants living outside their own communities, for example, studies based on inpatient rehabilitation or palliative care. Studies that include participants with and without stroke will be considered for inclusion if data pertaining to stroke survivors can be specifically extracted.

We specifically decided to include only individuals who are at least six months post-stroke as stroke is a life-altering medical condition, not only for the patient but also for their family members. ^26^ During the first six months, patients typically undergo multiple rehabilitative and medical appointments as they adjust to their new normal. ^27^ The factors impacting community ambulation during the acute to subacute stages may not be relevant for the rest of their lives as stroke survivors. ^28^ Six months post-stroke is considered the chronic phase, and from this stage onward, the factors affecting community ambulation may be influenced by multiple physical, personal, and psychosocial aspects, rather than just the acute effects of the stroke. ^29^ Therefore, we chose to map the factors associated with community ambulation only in individuals who are at least six months post-stroke.

### Concept

This review will consider studies that explore the concept of post-stroke community ambulation, defined as “independent mobility outside the home, which includes the ability to confidently negotiate uneven terrain, private venues, shopping centers and other public venues”. ^30^ We will include studies that specifically assess community ambulation, that is, walking outside one’s home, through daily stepping activity or self-report questionnaires, or predict community ambulator status based on clinical outcomes such as gait speed or the Six-Minute Walk Test (6 MWT). To ensure that self-report questionnaires focus on assessing true community ambulation, at least 25% of the questions or items on the scale must assess walking activity outside one’s home. ^23^ Studies will be excluded if they assess general physical activity levels that may not be related to walking outside one’s home such as swimming, yoga, or biking. We will also exclude studies that only assess ‘community ambulation’ through modes of mobility other than walking, such as driving, biking, or using public transportation.

### Context

This review will consider studies that quantitatively explore at least one physical (e.g., gait speed, walking endurance, balance), biopsychosocial (e.g., age, gender, race, living with spouse, self-efficacy, depression, use of assistive device), or environmental (e.g., neighborhood walkability, Area Deprivation Index) factor(s) that impact community ambulation post-stroke. Eligible studies will not be limited to any geographic location.

## Methods

This mapping review will consider quantitative study designs, such as cross-sectional, observational, case-control, prospective, and retrospective cohorts, and longitudinal studies for inclusion. In addition, any interventional (randomized or non-randomized) studies that assessed any factors associated with community ambulation at baseline measurement and mixed-methods studies will also be included in this review. Individual studies within evidence syntheses will be screened if they are not identified through the primary literature search. Qualitative studies will be excluded from the analysis owing to a lack of quantitative evidence of the association between any measured factors and community ambulation post-stroke, a primary aim of this mapping review. In mixed-methods studies, only the quantitative portion will be considered for inclusion. Case studies and case series will be excluded as they provide limited quantitative data and represent a lower level of evidence. Conference abstracts and master/PhD dissertations will be excluded unless they are subsequently published as peer-reviewed studies.

### Search strategy

A health sciences librarian (JMD) developed search strategies using research team input and conducted a literature search. The search strategy aimed to locate primary studies published in English. A test search between December 2018 and June 2019 using the CINAHL, PubMed, and PsycINFO databases was performed to identify articles on the topic. The text words contained in the titles and abstracts of relevant articles and the index terms used to describe these articles were used to develop a full search strategy that was peer-reviewed by another health sciences librarian. The following databases were selected for the final literature search because of their topic coverage in the health and social sciences: Applied Social Sciences Index and Abstracts (ProQuest), CINAHL (EBSCOHost), PEDro, Psychology and Behavioral Sciences Collection (EBSCOHost), PsycINFO (EBSCOHost), PubMed, REHABDATA (NARIC), SPORTDiscus (EBSCOHost), and Web of Science (Clarivate Analytics). During the final literature search conducted on July 14, 2023, keywords were truncated and phrase-searched, and the search strategy was translated for each database using controlled vocabulary whenever possible. The search strategy, including all the identified keywords and index terms, was adapted for each information source. The search strategy for PubMed is publicly available as Extended data ^31^ (see data availability statement), and all search strategies are available by contacting the lead author. The bibliographies of the articles included in the review will also be screened for additional papers not located in the literature search. Handsearching will not occur, as the selected bibliographic databases will cover all key journals (Clinical Rehabilitation, Journal of Neurologic Physical Therapy, Neurorehabilitation and Neural Repair, Physical Therapy, Physical Therapy Journal, and Topics in Stroke Rehabilitation). The search will be re-run before publishing the full mapping review to ensure that the most recent articles are retrieved.

Articles published on or after January 1, 2001, to the present will be included as the World Health Organization’s International Classification of Functioning, Disability, and Health, was published in 2001. During this time, healthcare professionals transitioned their focus from ‘impairment-based disease’ to ‘function-based health’. The ICF model provides a foundational framework for this mapping review as it focuses on the domains of body structure, body function, and personal factors that could affect activities and participation related to community walking.

### Study/Source of evidence selection

Following the updated literature search, all identified records will be collated and uploaded to Endnote Web [Clarivate Analytics ^TM^, PA, USA] and manually de-deduplicated in preparation for screening in Covidence [Melbourne, Australia]. Following a pilot test, titles and abstracts will be screened by any two out of three independent reviewers (KB, SAC, and DKR) to assess the inclusion criteria for the review. Potentially relevant papers will be retrieved in full, and their citation details will be automatically imported into Covidence [Melbourne, Australia]. The full texts of the selected citations will be assessed in detail against the inclusion criteria by any two of the three independent reviewers (KB, SAC, and DKR). The reasons for excluding full-text papers will be recorded and reported in the mapping review. Any disagreements between the reviewers at each stage of the selection process will be resolved through discussion or by the third reviewer. The results of the search will be reported in full in the final mapping review and will be presented in a PRISMA flow diagram. ^32^

### Data extraction

Data will be extracted from the papers included in the mapping review by two independent reviewers using a data extraction tool developed by the reviewers (see Extended data - Appendix II). ^31^ The extracted data will include specific details about participant characteristics, methods of quantifying community ambulation and the corresponding descriptive data, factors assessed and associated with community ambulation, and key findings relevant to the review questions. The draft data extraction tool will be modified and revised, if necessary, during the process of extracting data from each included paper. Modifications will be detailed in the full mapping review. Any disagreements between the reviewers will be resolved through discussion or by the third reviewer. Authors of papers will be contacted to request missing or additional data where required. The included studies will also be critically appraised using a quality assessment tool modified from the NIH Quality Assessment Tool for Observational Cohort and Cross-Sectional Studies (see Extended Data - Appendix III). ^31^ Critical appraisal will also be conducted by the two independent reviewers, and any conflicts will be resolved by the third reviewer.

### Data analysis and presentation

Extracted data will be presented in tabular format with a summary of the key findings from each included study. Descriptive statistics and frequency counts will be provided for the participant characteristics and tools used to assess community ambulation. To describe the extent of post-stroke community ambulation, quantifiable data will be pooled from studies that would use a homogeneous assessment tool, such as daily steps, gait speed, or community trips/day. Additionally, based on the extracted data, we will create an evidence map of all examined physical, biopsychosocial, and environmental factors affecting community ambulation post-stroke within the ICF framework. This evidence map will be color-coded to depict the importance of each factor in relation to community ambulation, as determined by the number of studies that analyzed the factors and their cumulative sample sizes. A narrative summary will accompany the tabulated and graphical results and will describe how the results relate to the review’s objectives and questions, followed by recommendations for future research based on the identified gaps in the literature.

## Discussion

This mapping review aims to report the physical, biopsychosocial, and environmental factors affecting community ambulation post-stroke. The evidence gained will be used to build an ICF-based model of community ambulation, which will in turn inform future interdisciplinary clinicians, policymakers, and rehabilitation scientists to develop meaningful interventions to improve community ambulation amongst stroke survivors. Additionally, this review will describe the various methods used to examine community ambulation and shed light on the extent to which individuals achieve community ambulation post-stroke. Overall, the mapped evidence will motivate future studies to develop holistic approaches that specifically focus on the most vital factors that may influence community ambulation and facilitate the recovery and reintegration of stroke survivors in their communities.

### Ethical considerations

Ethical approval was not required for this mapping review, as we will use secondary de-identified data.

### Dissemination

The results of this mapping review will be disseminated through national and international conference presentations as well as peer-reviewed, high-impact journal publications.

### Study status

This study is currently in the data extraction phase and will be followed by data analysis, synthesis, visualization, and drafting of the final manuscript.

## Data Availability

No data are associated with this article. Open Science Framework: Factors Affecting Community Ambulation Post-stroke: A Mapping Review Protocol (Extended Data), https://doi.org/10.17605/OSF.IO/SEMWQ. ^31^ This project contains the following extended data:
• Appendix I: Search strategy for PubMed, conducted on December 16, 2021 • Appendix II: Data extraction instrument • Appendix III: Quality Assessment Tool Data are available under the terms of the Creative Commons Attribution 4.0 International license (CC-BY 4.0).
