# Supplementary material for: Factors affecting community ambulation post-stroke: a mapping review protocol
Source: F1000Res. 2024 Aug 29;13:166. Originally published 2024 Mar 8. [Version 2] doi: 10.12688/f1000research.144582.2 (PMC11364963; doi:10.12688/f1000research.144582.2)
Supplement: Peer review history

# Reviewer report: Reviewer response for version 2

**DOI:** 10.5256/f1000research.170984.r318624

**Published:** 2024-08-30

**Authors:** Ahmad Sahely

**Affiliations:** 1 University of Birmingham, Birmingham, UK; 2 Physical Therapy Department, Jazan University, Jazan, Jazan, Saudi Arabia

The authors have addressed my comments. It is good to go for indexing.

Is the study design appropriate for the research question?

Yes

Is the rationale for, and objectives of, the study clearly described?

Yes

Are sufficient details of the methods provided to allow replication by others?

Yes

Are the datasets clearly presented in a useable and accessible format?

Not applicable

Reviewer Expertise:

Stroke rehabilitation, mobility, community, mixed methods

I confirm that I have read this submission and believe that I have an appropriate level of expertise to confirm that it is of an acceptable scientific standard.

# Reviewer report: Reviewer response for version 1

**DOI:** 10.5256/f1000research.158396.r299999

**Published:** 2024-07-23

**Authors:** Ahmad Sahely

**Affiliations:** 1 University of Birmingham, Birmingham, UK; 2 Physical Therapy Department, Jazan University, Jazan, Jazan, Saudi Arabia

The topic being discussed in the review is very important to inform future research in the field about factors affecting stroke survivors' ambulation in the community. The stated rationale is reasonable to reviewing current literature and synthesise evidence that can enlighten future interventions. 

 Here are some comments to consider:

 - Is there any reason for including only individuals who are at least 6 months post-stroke, there might be some eligible participants less than 6 months with experience of ambulation in the community after discharge for sub-acute care.

 - The study cited down provides some perspectives on the topic. You might include it in you review if eligible or discuss factors affecting ambulation in the community in the discussion section.

 - Consider inclusion of qualitative/mixed methods studies as they can provide rich data from patients' perspectives about their experience of ambulation in the community.

Is the study design appropriate for the research question?

Yes

Is the rationale for, and objectives of, the study clearly described?

Yes

Are sufficient details of the methods provided to allow replication by others?

Yes

Are the datasets clearly presented in a useable and accessible format?

Not applicable

Reviewer Expertise:

Stroke rehabilitation, mobility, community, mixed methods

I confirm that I have read this submission and believe that I have an appropriate level of expertise to confirm that it is of an acceptable scientific standard, however I have significant reservations, as outlined above.

## References

[1] : Self-management interventions to improve mobility after stroke: an integrative review. *Disabil Rehabil* .2023;45(1) : 10.1080/09638288.2022.2028019 9-26 10.1080/09638288.2022.2028019 35068313

# Response

**Published:** 2024-08-19

**Authors:** Kanika Bansal

**Affiliations:** Physical Therapy, University of Mount Union, Alliance, Ohio, USA

**Author response to reviewer comments:**

**Comment:** *Is there any reason for including only individuals who are at least 6 months post-stroke? There might be some eligible participants less than 6 months with experience of ambulation in the community after discharge from sub-acute care.*

**Response:** Thank you for this comment. We decided to include only individuals who are at least 6 months post-stroke because stroke is a life-altering medical condition, not only for the patient but also for their family members. ^1^ During the first six months, patients typically undergo multiple rehabilitative and medical appointments as they adjust to their new normal. ^2^ The factors impacting community ambulation during the acute to subacute stages may not be relevant for the rest of their lives as stroke survivors. ^3^ Six months post-stroke is considered the chronic phase, and from this stage onward, the factors affecting community ambulation may be influenced by multiple physical, personal, and psychosocial aspects, rather than just the acute effects of the stroke. ^4^ Therefore, we chose to examine the factors associated with community ambulation only in individuals who are at least 6 months post-stroke.

**Comment:** *The study cited below provides some perspectives on the topic. You might include it in your review if eligible or discuss factors affecting ambulation in the community in the discussion section.*

*(* *https://www.tandfonline.com/doi/full/10.1080/09638288.2022.2028019#abstract* *)*

**Response:** Thank you for sharing this impressive research. We will certainly consider including it in our discussion of the full mapping review. I agree that self-management is key to long-term and sustainable improvement in mobility post-stroke.

**Comment:** *Consider inclusion of qualitative/mixed methods studies as they can provide rich data from patients' perspectives about their experience of ambulation in the community.*

**Response:** Thank you for bringing this point up. We agree that patients’ perspectives on their experiences with community ambulation can provide valuable information to clinicians and stakeholders. However, our aim was to present the factors examined in community ambulation post-stroke specifically in studies that measured community ambulation through clinical outcomes, daily steps, or questionnaires. As qualitative studies may or may not measure community ambulation, many would be excluded based on our criteria. Furthermore, we plan to present the most commonly examined factors affecting community ambulation in a ‘traffic light’ format that highlights the frequency of each factor. This will include the number of studies that examined the factor and the total number of individuals for whom the factor was or was not statistically associated with community ambulation, using a color scheme: green (frequently associated), orange (mixed evidence), and red (frequently not associated). ^5^ Given the aims of this mapping review, including qualitative studies may not do justice to the data presented. Nonetheless, we will acknowledge the lack of inclusion of qualitative studies as a limitation in our full mapping review. We will also consider conducting a separate qualitative mapping review in the future to specifically address patient perspectives on community mobility post-stroke, which may complement our current quantitative mapping review.

**References used in the response:**

 1.           Lutz BJ, Young ME, Cox KJ, Martz C, Creasy KR. The Crisis of Stroke: Experiences of Patients and Their Family Caregivers. *Top Stroke Rehabil*. 2011;18:10.1310/tsr1806-786.

 2.           Ances BM, D’esposito M. Review Article: Neuroimaging of Recovery of Function after Stroke: Implications for Rehabilitation. *Neurorehabil Neural Repair*. 2000;14:171-179.

 3.           Mahendran N, Kuys SS, Brauer SG. Recovery of ambulation activity across the first six months post-stroke. *Gait Posture*. 2016;49:271-276.

 4.           Grefkes C, Fink GR. Recovery from stroke: current concepts and future perspectives. *Neurol Res Pract*. 2020;2:17.

 5.           Novak I, Morgan C, Fahey M, et al. State of the Evidence Traffic Lights 2019: Systematic Review of Interventions for Preventing and Treating Children with Cerebral Palsy. *Curr Neurol Neurosci Rep*. 2020;20:3.

# Reviewer report: Reviewer response for version 1

**DOI:** 10.5256/f1000research.158396.r275040

**Published:** 2024-05-29

**Authors:** Timothy Hanke

**Affiliations:** 1 Midwestern University, Downers Grove, Illinois, USA

The authors intend to conduct a scoping (mapping) review with the purpose to report the physical, biopsychosocial, and environmental factors affecting community ambulation in persons' post-stroke. The topic is timely, relevant, and there will be an interested readership from any number of healthcare professions. 

 The introduction is well-written, logical, and provides sufficient evidence for the need for this mapping review. 

 The study design is robust and includes the relevant methodology. The search strategy, databases/search terms are appropriate. Whether or not the grey literature is being used was addressed. The data extraction process is described. Covidence is being used. There are safeguards by using pairs of reviewers and a third for addressing any discrepancies. Critical appraisal will be conducted. As such, methods are adequately described.

Is the study design appropriate for the research question?

Yes

Is the rationale for, and objectives of, the study clearly described?

Yes

Are sufficient details of the methods provided to allow replication by others?

Yes

Are the datasets clearly presented in a useable and accessible format?

Not applicable

Reviewer Expertise:

Community mobility in persons' post-stroke; balance and falls in community-dwelling older adults.

I confirm that I have read this submission and believe that I have an appropriate level of expertise to confirm that it is of an acceptable scientific standard.
